# Functional analysis of the *Escherichia coli mrdA* gene in melittin resistance

**DOI:** 10.3389/fmicb.2024.1516808

**Published:** 2025-03-05

**Authors:** Chong-Yi Zhao, Xiao Li, Ting Zhao, Ying Liu, Xue-Shan Xia, Xiao-Mei Wu

**Affiliations:** ^1^Department of Gynecology, The First People’s Hospital of Yunnan Province, The Affiliated Hospital of Kunming University of Science and Technology, Kunming, China; ^2^Medical School, Kunming University of Science and Technology, Kunming, China; ^3^Faculty of Life Science and Technology, Kunming University of Science and Technology, Kunming, China

**Keywords:** antimicrobial peptide, melittin, *mrdA* gene, resistance, transpeptidase

## Abstract

**Objective:**

The aim of this study is to examine the functional role and resistance mechanisms of the *Escherichia coli* (*E. coli*) peptidoglycan transpeptidase gene, *mrdA*, in resistance to melittin.

**Methods:**

The resistance of *E. coli* strains with either knockout or overexpression of the *mrdA* gene to melittin was initially assessed. The differences in melittin absorption between these two strains were evaluated following depletion and heterologous expression of the *mrdA* gene. Subsequently, peptidoglycan was extracted from the strains to determine its capacity to adsorb melittin. Finally, the morphological changes in different strains induced by melittin exposure were examined under scanning electron microscopy. These analyses served to validate the role of peptidoglycan transpeptidase *mrdA* in melittin resistance and to hypothesize its potential resistance mechanism.

**Results:**

The results clearly indicated a direct correlation between the degree of peptidoglycan cross-linking in *E. coli* and its enhanced resistance to melittin. Specifically, we found that increased cross-linking of peptidoglycan led to a thickening of the bacterial cell wall and a reduction in pore size. These structural changes potentially decrease the damage to the cell wall caused by melittin, as the thicker cell wall and smaller pores reduce the ability of melittin to penetrate and access the interior of bacterial cells. This mechanism effectively limits the contact between melittin and bacterial components, minimizing its destructive effects, and thereby conferring resistance to melittin in the bacteria.

**Conclusion:**

This study is the first to elucidate the role of peptidoglycan in the cell wall of *E. coli* in the context of antimicrobial peptide resistance. Novel ideas have been proposed for the development of antibacterial drugs targeting the peptidoglycan of Gram-negative bacteria.

## 1 Introduction

The growing resistance to antibiotics has emerged as a concern worldwide, underscoring the need for the development and use of new antibacterial drugs. Antimicrobial peptides, characterized by short peptide chains, exhibit bactericidal activity through diverse mechanisms and hold significant potential for therapeutic applications (Mousavi Maleki et al., 2023). However, as the use of antimicrobial peptides becomes widespread, the challenges posed by drug resistance have become increasingly prominent. Colistin-resistant *Escherichia coli* strains have been identified even in the intestines of humans and wildlife that have not been directly exposed to colistin (Torres et al., 2021; Olaitan et al., 2015). The problem is further compounded by the emergence of resistance associated with antimicrobial peptides (Samuelsen et al., 2005; Roland et al., 1993), combinations of antimicrobial peptides and antibiotics (Blanco et al., 2020), and cross-resistance between antimicrobial peptides and host defense peptides (Kubicek-Sutherland et al., 2016; Habets and Brockhurst, 2012). Therefore, it is essential to examine the mechanisms of antimicrobial peptide resistance and develop strategies to prevent or delay its onset.

Peptidoglycan is an important structural component of the bacterial cell wall and serves as both the target of action and defense mechanism against numerous antibacterial agents. Drugs that exert bactericidal effects by interfering with peptidoglycan biosynthesis and cross-linking mainly include β-lactam antibiotics, glycopeptide antibiotics, and certain antimicrobial peptides (Hsu et al., 2004; Brötz et al., 1998; Neelay et al., 2017). In terms of resistance, methicillin-resistant *Staphylococcus aureus* (MRSA) alters penicillin-binding proteins (PBPs) through gene mutations or horizontal gene transfer, thereby reducing their affinity for β-lactam antibiotics (Fisher et al., 2005). Similarly, vancomycin-resistant enterococci (VRE) circumvent peptidoglycan cross-linking inhibition by modifying the D-alanyl-D-alanine dipeptide in peptidoglycan precursors to D-alanyl-D-lactate (Arthur and Courvalin, 1993). The lipid II structure is modified by bacteria such as nisin-resistant strains to limit the interaction between nisin and its target (Guilhelmelli et al., 2013; Hébert et al., 2007). In addition, certain Gram-positive bacteria may also decrease antibiotic penetration by increasing the thickness of the peptidoglycan layer (Fisher et al., 2005; Arthur and Courvalin, 1993).

Compared to Gram-positive bacteria, the peptidoglycan layer in the cell wall of Gram-negative bacteria is considerably thinner, with the former ranging from 20–80 nm in thickness while the latter measures only 7.5–10 nm (Alkasher and Badi, 2020). Due to this relatively thin peptidoglycan layer, studies on the role of this structure in the resistance of Gram-negative bacteria are limited. In previous research (Zhao et al., 2024), a melittin-resistant *E. coli* peptidoglycan transpeptidase encoding gene (*mrdA*, UniProt ID: P0AD65) was identified through functional metagenomics in soil and human intestinal samples as part of an antimicrobial peptide resistance screening panel. In order to further explore its function and resistance mechanism, the relationship between peptidoglycan cross-linking during peptidoglycan synthesis and melittin resistance in *E. coli* and the related mechanisms were analyzed in this study. The role of peptidoglycan in conferring resistance to antimicrobial peptides in the *E. coli* cell wall was revealed for the first time, offering new perspectives for the development of antibacterial agents using peptidoglycan as an antibacterial target.

## 2 Materials and methods

### 2.1 Construction of the *mrdA* knockout mutant in the *Escherichia coli* MG1655 strain

Knockout primers (mrdA-F, mrdA-R), identification primers (mrdA-R1), and gRNAs were designed using Snapgene, based on the *mrdA* gene sequence (GenBank: X04516.1) (Supplementary Table 1). Bacterial genomes were edited using the CRISPR-Cas system in conjunction with the Red recombination system. Knockout positive clones (∆*mrdA*) were successfully obtained.

### 2.2 Growth curve analysis of the *mrdA* mutant in the *Escherichia coli* MG1655 strain

Prior to experimentation, 96-well plate covers were treated with a solution of 9 mL absolute ethanol and 1 mL Triton X-100 for 15 s (Brewster, 2003), placed under a UV lamp in a laminar flow hood for 15 min for ventilation and drying. Overnight cultures of both the *E. coli* MG1655 *mrdA* mutant and wild-type strains were diluted to a final concentration of OD600 = 0.01 in Luria–Bertani (LB) broth and transferred into the prepared 96-well plates, with four replicates for each strain. The plates were then sealed with parafilm to prevent evaporation. Growth curves were measured using a microplate reader under the ABS kinetic method, with readings taken at 600 nm (OD600) every hour for 15 h at a constant temperature of 37°C. Each well was shaken circularly for 3 s before every reading.

### 2.3 Determination of antimicrobial peptide resistance in *mrdA* mutants of the *Escherichia coli* MG1655 strain

Minimal inhibitory concentrations (MICs) of melittin were determined for both *E. coli* MG1655 *mrdA* knockout and wild-type strains using Mueller–Hinton (MH) and LB media (Wiegand et al., 2008). Wild-type and mutant strains were cultured in LB medium to the stationary phase, and the optical density (OD) was uniformly adjusted to 1.0. Melittin was added to a final concentration of 20 μg/mL and placed in a shaker at 37°C and 220 rpm for 2 h. Bacterial cultures were serially diluted across six gradients (10-fold dilutions), and 10 μL of each dilution was spotted onto LB agar plates. After 8 h of inverted incubation at 37°C, bacterial growth was observed to assess melittin resistance.

### 2.4 Heterologous expression of the *mrdA* gene in the *Escherichia coli* BL21 (DE3) strain

The pET-28a (+) plasmid was employed as the expression vector, using *EcoRI* and *SalI* as the restriction enzyme sites. Primers containing homologous recombination arms and CDS amplification primers were designed using Snapgene (Supplementary Table 2). The *E. coli* BL21 (DE3) strain was used as a host to successfully construct an overexpression strain of *mrd*A (designated as *mrd*A-OE). Additionally, a pET-28a (+) empty vector was transformed into *E. coli* BL21 (DE3) as a control strain (vector).

Cultures were grown in self-induction medium (Supplementary Tables 3, 4) (Studier, 2014), and target protein expression was assessed at OD600 values of 0.4, 0.6, 0.8, and 1.0. For each time point, 100 mL of bacterial culture was collected, centrifuged at 8,000 rpm for 5 min at 4°C. The supernatant was discarded, and the pellet was resuspended in 10 mL of ice-cold phosphate-buffered saline (PBS) buffer. After repeated centrifugation, the pellet was further resuspended in 1 mL of ice-cold ultrasonic buffer (Supplementary Table 5) and thoroughly mixed. The bacterial suspension was sonicated using an saline ice bath for ultrasonic rupture of bacteria, with ultrasonic parameters set to 400 W, working for 5 s and with 6-s intervals, for a total of 20 min. Subsequently, centrifugation was performed at 14,000 rpm for 40 min at 4°C to separate the supernatant and pellet. The pellet was suspended in 800 μL PBS, and total protein concentrations in both the supernatant and pellet were measured using the bicinchoninic acid assay (BCA) method. Protein loading was standardized to 40 μg, with a loading volume of 20 μL. Samples were incubated with 5× protein loading buffer in a 95°C-water bath for 5 min, followed by centrifugation at 12,000 rpm for 1 min. Protein expression was quantified using 10% sodium dodecyl-sulfate polyacrylamide gel electrophoresis (SDS-PAGE) with Coomassie blue staining and western blotting (WB). WB was used to detect protein expression (Supplementary Table 5).

### 2.5 Determination of melittin resistance in *mrdA*-overexpressing *Escherichia coli*

The MICs of melittin were determined for *E. coli* strains overexpressing *mrdA*. Spot plate experiments were performed by exposing the *mrdA*-overexpressing *E. coli* strain to melittin for 2 h. Simultaneously, 5 μL of the *mrdA*-overexpressing strain and the control strain (cryopreserved at −80°C) were inoculated into 5 mL of LB medium containing kanamycin (Kan) at a dilution of 1:1000 and cultured at 37°C for 12 h on a shaker at 200 rpm.

The bacterial suspensions were then diluted from the induction medium to a final concentration of OD600 = 0.01 and transferred into 96-well plates. A plate reader was used to measure OD600 at a constant temperature of 37°C, with readings taken every hour for a total of 6 h. Upon reaching an OD600 of 0.6, melittin at 4 times the MIC concentration was added to the wells, and measurements were continued for an additional 13 h using the plate reader.

### 2.6 High-performance liquid chromatography detection of melittin content in bacterial cells

Mutant and wild-type *E. coli* strains were cultured in LB medium for 12 h overnight, while bacterial cultures containing either the empty vector or *mrdA*-overexpressed strains were grown in autoinduction medium for the same duration. All bacterial cultures were adjusted to an OD of 1.0 in a uniform volume of 150 mL. Melittin was then added at a final concentration of 20 μg/mL, and the cultures were incubated at 37°C with shaking at 200 rpm on a shaker for 2 h. The OD was readjusted to 1.0, and the final volume of each culture was standardized to 100 mL.

The cells were harvested by centrifugation at 8,000 rpm for 5 min at 4°C, and the wet bacterial pellet was weighed. The pellet was washed four times with PBS and suspended in 5 mL of ice-cold PBS. The bacteria were ruptured using sonication at 4°C and the resulting suspension was centrifuged at 14,000 rpm for 40 min. The isolated supernatant was filtered through a 0.22-μm filter membrane and stored at −40°C for subsequent analysis. Parameters for high-performance liquid chromatography (HPLC) are shown in Supplementary Table 6.

### 2.7 Experiments on the adsorption of melittin by peptidoglycan

Reagents and solutions used in this experiment were prepared as shown in Supplementary Table 7. Peptidoglycan sacculus were extracted from each strain following the classical method described by Schaub and Dillard (2017). Strains were cultured to stationary phase in 2,000 mL of LB medium, and the pellets were harvested by centrifugation at 6,000 × g for 10 min at 4°C. Each strain’s pellet was uniformly weighed to 4 g, resuspended in 10 mL of PB solution, and added dropwise to an equal volume of boiling PB buffer containing 8% SDS (w/v). This mixture was further boiled for 1 h.

After boiling, the suspension was cooled to room temperature and placed on a shaker at 220 rpm overnight at 37°C. The suspension was then centrifuged at 130,000 × g for 30 min at 15°C. The supernatant was discarded, and the pellet was washed multiple times with PB solution, followed by centrifugation until the SDS was fully removed. The resulting pellet was the extracted peptidoglycan “sacculus,” which was weighed and documented.

A 1 mg/mL solution of the peptidoglycan in PBS was prepared, and melittin was added to a final concentration of 20 μg/mL. This mixture was placed on a shaker at 37°C with a speed of 220 rpm for 3 h. After the reaction, the suspension was centrifuged at 130,000 × g for 30 min, and the supernatant was collected for HPLC assay analysis. The amount of melittin adsorption capacity of the extracted peptidoglycan from different strains (intracellular melittin, or GQ) was calculated using the following formula: Adsorption = (amount of GQ added − amount of GQ in supernatant)/weight of peptidoglycan (mg).

### 2.8 Morphological analysis of the effect of melittin on *Escherichia coli* using scanning electron microscope

*E. coli* cultures were prepared by adding 5 mL of LB medium to the cells, followed by incubation at 37°C with shaking at 200 rpm until the OD reached 1.0. After routine culturing, the bacterial suspension was centrifuged and the supernatant was discarded. The pellet was then fixed with a Gluta fixative (2.5% glutaraldehyde, v/v) at a volume 40 times that of the bacterial pellet, mixed thoroughly, and stored at 4°C for 4 h. After fixation, the suspension was centrifuged at 6,000 rpm for 5 min, and the supernatant was discarded. The pellet was washed three times with distilled water, centrifuged, and the supernatant was discarded.

Dehydration was carried out using a gradient of ethanol concentrations of 30, 50, 70, 80, and 90%, with each step lasting 15 min, and the samples were centrifuged at 6,000 rpm for 5 min between each step. The samples were further dehydrated in 100% ethanol for 15 min twice. Following this, the samples were placed in a 1:1 mixture of ethanol and tert-butanol for 20 min and then replaced with 100% tert-butanol twice for 20 min each. After the final replacement, the sample containing a small amount of tert-butanol was pre-cooled and then dried under vacuum in a freeze-dryer precooled at −10°C for 12 h. Once dehydration was complete, the samples were sputter-coated with a gold layer to produce a 5-nm-thick conductive film and observed under a scanning electron microscope (SEM).

Both wild-type and *mrdA* mutant strains were routinely cultured, with the *mrdA*-overexpressing strains grown in a self-induction medium. After adjusting the OD of each bacterial solution to 1.0, 1 mL of the culture was centrifuged at room temperature for 5 min at 6,000 rpm. The bacterial pellets were suspended in 300 μL of culture medium. Melittin was added to a final concentration of 20 μg/mL, and the cultures were incubated at 37°C and 200 rpm for 2 h.

Following incubation, 20 μL of each bacterial solution was pipetted onto 1 cm × 1 cm glass coverslips and allowed to dry at room temperature. The dried samples were dehydrated and processed as described above and sputter-coated with gold. The samples were observed under a scanning electron microscope.

For morphological analysis, eight cells were randomly selected from each SEM field of view and measured using the SEM’s built-in software. The data were analyzed and plotted using GraphPad software. SEM images were randomly selected for further examination. The total number of cells and the number of intact cells were counted based on the criteria for cell rupture and loss of cell contents. The proportion of intact cells in each figure was calculated as: the number of intact cells/the total number of cells in the field of view.

### 2.9 Statistical analysis

Growth curve comparison: The differences in growth curves between different strains were analyzed using repeated measures two-way analysis of variance (ANOVA). A *p*-value of <0.05 was considered indicative of significant differences in growth between strains.

Antimicrobial peptide resistance comparison: To compare the antimicrobial peptide resistance between overexpressing strains (*mrdA*-OE) and unloaded strains (vector) at different time points, the following calculations were performed:

OD600 values of *E. coli* overexpression strains without antimicrobial peptide treatment (*mrdA*-OE) (a) minus OD600 values of *E. coli* unloaded transformed strains (vector) at corresponding time points (b), OD600 values of *mrdA*-OE treated with antimicrobial peptide (a′) minus vector OD600 values at corresponding time points (b′), and a − b values at different time points were normalized by (a′ − b′)/(a − b) to compare antimicrobial peptide resistance between *mrdA*-overexpression strains and unloaded strains. A ratio >1 was considered evidence of resistance to the antimicrobial peptide in the *mrdA*-overexpression strains.

Determination of statistically significant differences: ns (not significant): *p* > 0.05; *: *p* ≤ 0.05; **: *p* ≤ 0.01; ***: *p* ≤ 0.001; ****: *p* ≤ 0.0001.

## 3 Results

### 3.1 *mrdA* knockout *Escherichia coli* exhibited increased sensitivity to melittin compared to wild-type *Escherichia coli*

**Figure 1:**
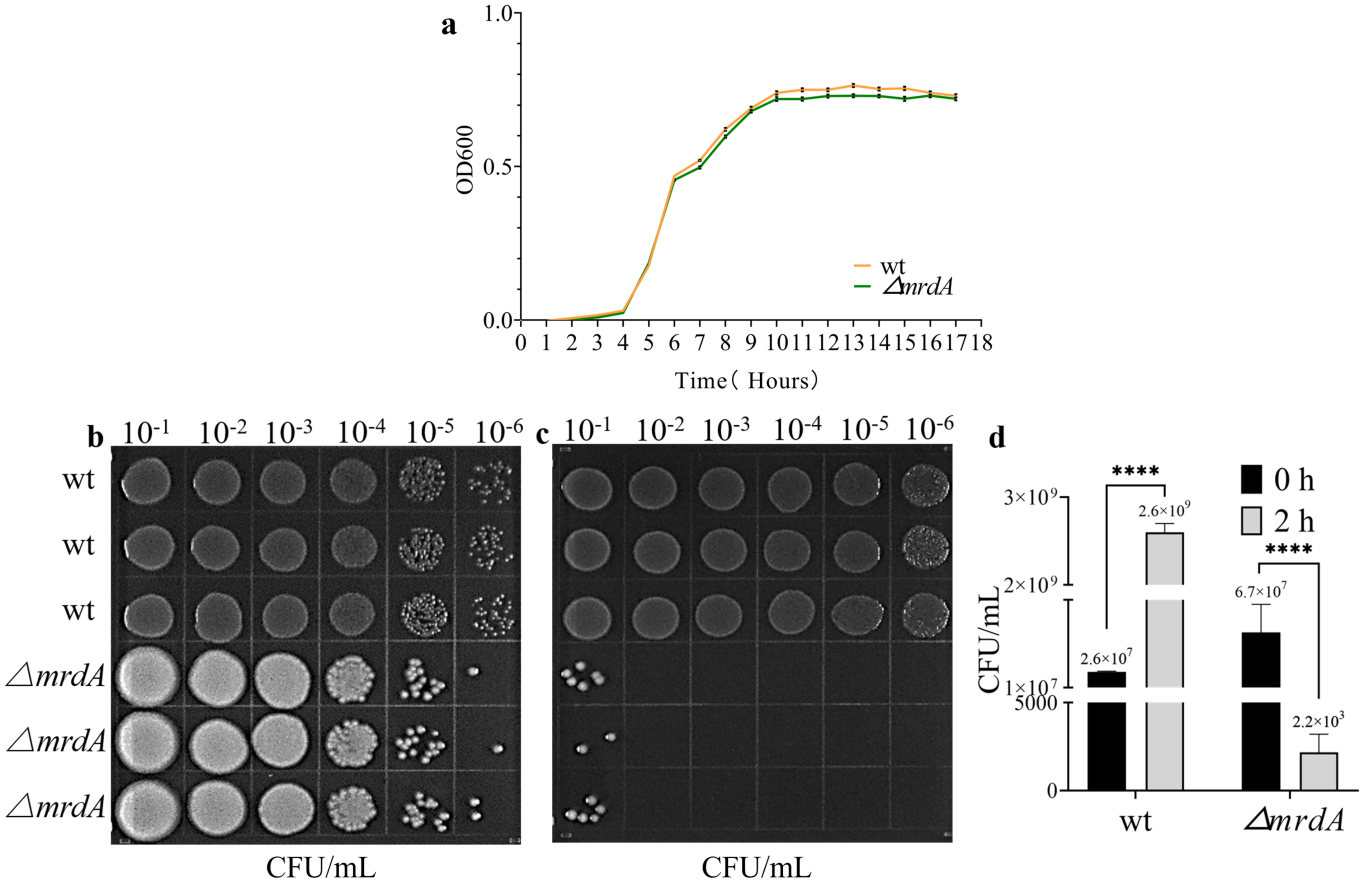
Growth curve of wild-type (wt) and ∆*mrdA* gene knockout (∆*mrdA*) *E. coli* strains, and the analysis of their resistance to melittin. **(a)** Growth curves of wt and ∆*mrdA* strains in LB medium over time (*n* = 3). **(b)** Survival of wt and ∆*mrdA* strains immediately after treatment with 20 μg/mL melittin (0 h, *n* = 3). Serial 10-fold dilutions (six gradients) were spotted (10 μL each) onto LB agar plates. **(c)** Survival of wt and ∆*mrdA* strains after 2 h of treatment with 20 μg/mL melittin (*n* = 3). Serial 10-fold dilutions (six gradients) were spotted (10 μL each) onto LB agar plates. **(d)** Colony count comparison between plates in panels b and c.

The growth trend of *mrdA* knockout *E. coli* (∆*mrdA*) was not significantly different from that of wild-type *E. coli* (wt) in LB medium (*p*-value = 0.0812, Figure 1a). Furthermore, there was no significant difference in growth between the two strains at different time points (Figure 1a). The results confirmed that deletion of the *mrdA* gene did not impact the growth of the *E. coli* MG1655 strain.

The findings from our melittin resistance testing have provided intriguing insights into the role of the *mrdA* gene in *E. coli*’s defense against melittin, a potent antimicrobial peptide. Our data revealed a stark difference in the minimal inhibitory concentration (MIC) of melittin required to inhibit the growth of the wild-type and the *mrdA* knockout (∆*mrdA*) strains of *E. coli*. Specifically, the wild-type strain demonstrated a higher tolerance to melittin, with an MIC of 32 μg/mL, indicating that it could withstand relatively higher concentrations of melittin without its growth being significantly impeded. In contrast, the ∆*mrdA* strain exhibited a considerably lower MIC of 8 μg/mL, suggesting that it is more susceptible to the antimicrobial effects of melittin. This marked increase in sensitivity in the absence of the *mrdA* gene underscores the gene’s potential role in conferring resistance to melittin. The substantial reduction in the MIC for the ∆*mrdA* strain implies that the *mrdA* gene may play a crucial part in the bacterial cell’s defense mechanisms against melittin, possibly by modulating the cell’s outer structure to impede melittin’s access to its targets within the cell.

Spot plate experiments additionally confirmed that the wild-type strain grew over time despite exposure to melittin at a final concentration of 20 μg/mL. Colony numbers were 100-fold higher after 2 h of melittin treatment compared to 0 h. ∆*mrdA*, on the other hand, showed a significant numerical reduction, with colony numbers decreasing to 3 × 10^−4^ after melittin treatment for 2 h compared to 0 h (Figures 1b–d).

### 3.2 *mrdA*-overexpressing *Escherichia coli* was resistant to melittin

Before induction, the MIC values for melittin were the same for both *E. coli* containing the empty vector (vector) and the *mrdA*-overexpressing strain (*mrdA*-OE), with a value of 4 μg/mL in LB medium.

**Figure 2:**
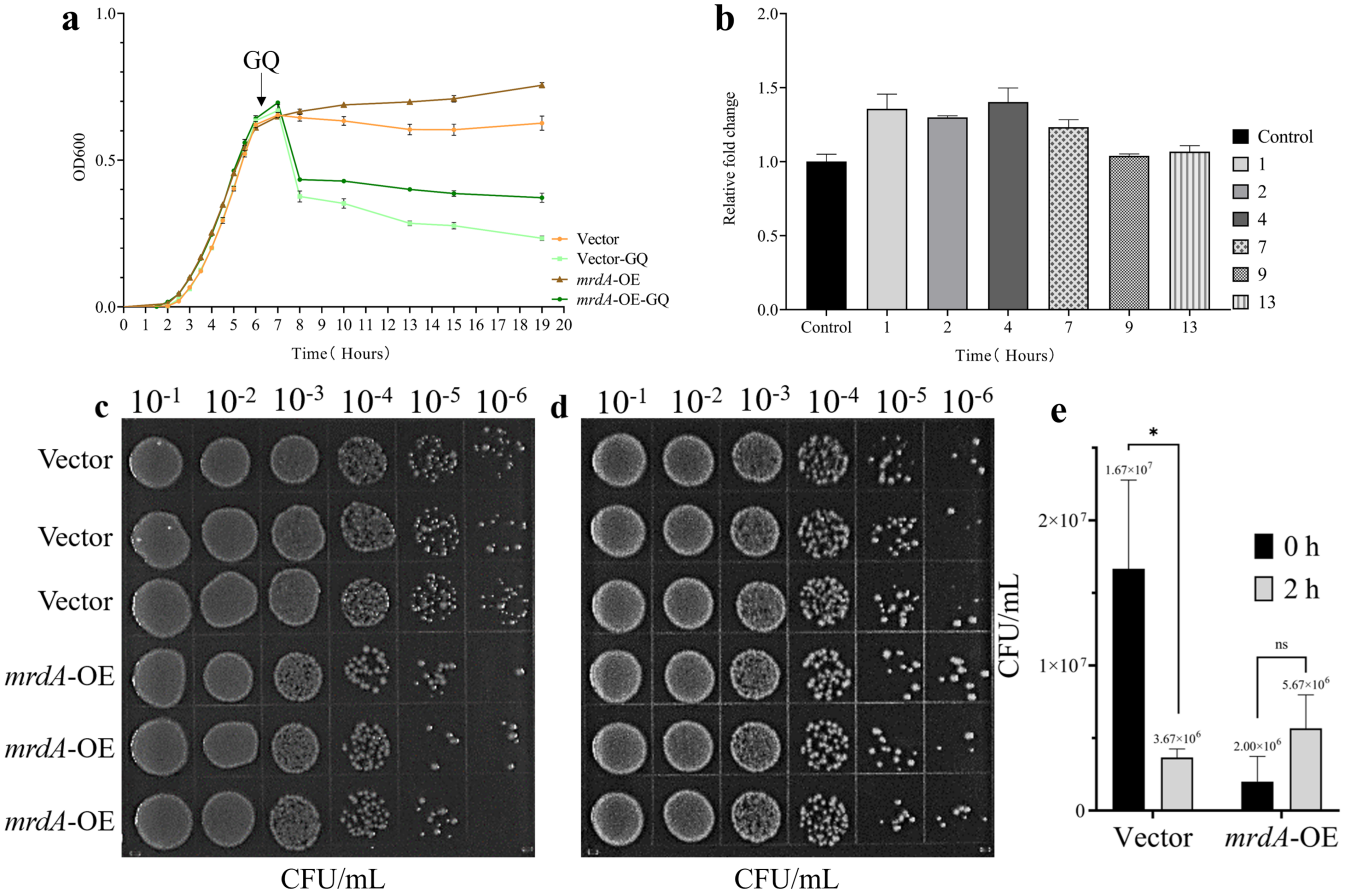
Analysis of melittin resistance in *E. coli* with vector and *mrdA* overexpression (*mrdA*-OE). **(a)** Growth curves of vector and *mrdA*-OE strains under 4× MIC of melittin (*n* = 4). **(b)** Resistance analysis of vector and *mrdA*-OE strains at different time points (1, 2, 4, 7, 9, and 13 h) after melittin treatment. A resistance value greater than 1 indicates resistance to melittin (*n* = 4). **(c)** Growth of vector and *mrdA*-OE strains immediately after treatment with 20 μg/mL of melittin (0 h) (*n* = 3). **(d)** Growth of vector and *mrdA*-OE strains after 2 h of treatment with 20 μg/mL of melittin (*n* = 3). **(e)** Colony count comparison between plates in panels **(c,d)**.

After induction in the specialized medium, vector and *mrdA*-OE showed different degrees of reduction with increasing antimicrobial peptide treatment time. The *mrdA*-OE strain showed stable resistance to melittin (Figures 2a,b), with an average resistance that was 1.23-fold higher than the vector strain (Figure 2b, mean resistance at each time point after melittin treatment), calculated from OD values at various time points.

Spot plate results further confirmed these observations, showing that, after melittin for 2 h, the number of colonies in the vector strain decreased approximately 5-fold compared to the initial count at 0 h, whereas *mrdA-*OE colonies increased 5.6-fold, exhibiting enhanced resistance to melittin (Figures 2c–e).

### 3.3 Analysis of differences in melittin absorption between *mrdA* mutants and overexpressing strains

**Figure 3:**
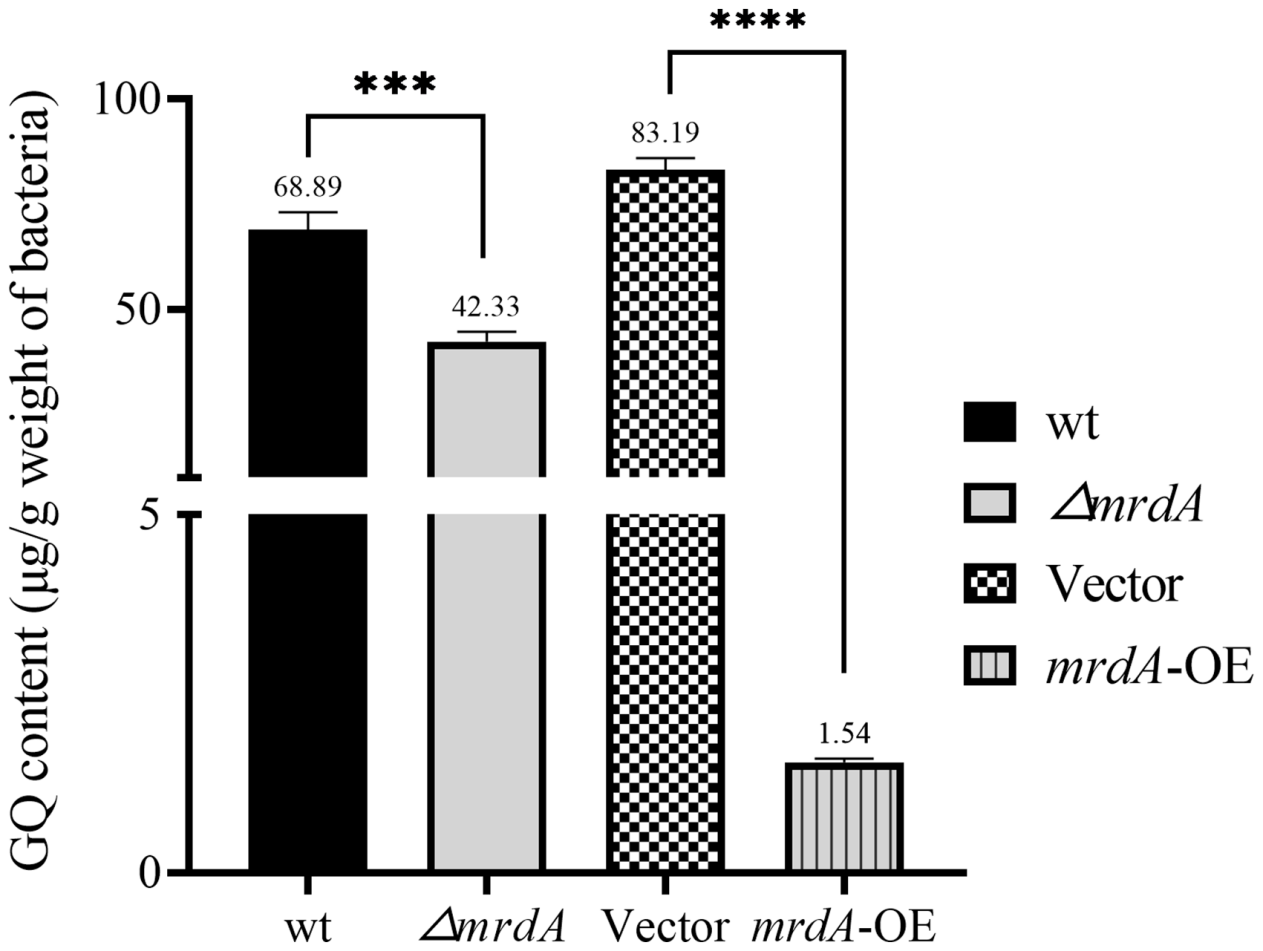
Quantification of intracellular melittin (GQ) in different strains after 2 h of treatment using HPLC (*n* = 3).

After 2 h of melittin treatment, the intracellular melittin content of wild-type (wt), *mrdA* knockout (∆*mrdA*), vector, and *mrdA*-overexpressing (*mrdA*-OE) strains was quantified using HPLC. The results showed that the intracellular melittin content per gram in the wt, ∆*mrdA*, vector, and *mrdA*-OE strains was 68.89 μg, 42.33 μg, 83.19 μg, and 1.54 μg, respectively. ∆*mrdA* intracellular melittin content was 61.45% of that in the wt strain, which was significantly different (*p* = 0.0008). The intracellular melittin content of *mrdA*-OE was only 1.85% of that of vector, and this difference was also significant (*p* < 0.0001) (Figure 3).

It is hypothesized that *mrdA*-overexpression increases the degree of peptidoglycan cross-linking and peptidoglycan thickness in host bacteria, thereby significantly restricting the entry of melittin into cells. Conversely, the lower melittin content in ∆*mrdA* mutant compared to the wt strain may be due to the extensive cell death of ∆*mrdA* observed after 2 h of melittin exposure (reflected by the 30,000-fold reduction in colony-forming unit after treatment). The high level of cell death likely resulted in a significant amount of melittin being bound to the remnants of dead cells, thereby depleting melittin in the medium, resulting in a lower intracellular concentration decrease in the surviving mutant cells.

### 3.4 Sorption analysis of melittin by peptidoglycan from *mrdA* mutants and *mrdA*-overexpressing strains

**Figure 4:**
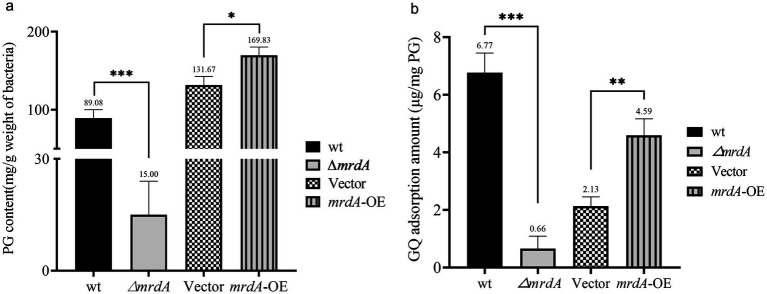
Comparative analysis of peptidoglycan (PG) content and melittin adsorption capacity among different strains. **(a)** Analysis of the differences in PG content extracted from wild-type (wt), ∆*mrdA*, vector control, and *mrdA*-OE strains (*n* = 3). **(b)** Comparative analysis of the melittin adsorption capacity of PG among the four strains (*n* = 3).

The results of the sorption analysis showed that peptidoglycan content in the *E. coli* cell wall was significantly related to *mrdA* expression. The peptidoglycan (PG) content extracted per gram of bacterial cells was 89.08 μg, 15.00 μg, 131.67 μg, and 169.83 μg for wt, ∆*mrdA*, vector, and *mrdA*-OE strains, respectively. The peptidoglycan content in ∆*mrdA* was 16.84% of that in the wt strain, and the difference was significant (*p* = 0.0008). The peptidoglycan content in *mrdA-*OE was 1.29-fold higher than vector and this difference was significant (*p* = 0.0122) (Figure 4a).

The ability of peptidoglycan to adsorb melittin was examined after 3 h of treatment. The results showed that this ability was related to the degree of cross-linking of peptidoglycan. The amount of melittin adsorbed per mg of extracted peptidoglycan was 6.77 μg, 0.66 μg, 2.13 μg, and 4.59 μg for wt, ∆*mrdA*, vector, and *mrdA*-OE strains, respectively. ∆*mrdA* peptidoglycan showed 9.75% adsorption capacity for melittin compared to the wt strain, which was significantly different (*p* = 0.0002). The adsorption capacity of *mrdA*-OE peptidoglycan for melittin was 2.14-fold higher than that of vector, and this difference was significant (*p* = 0.0029) (Figure 4b).

### 3.5 Effect of melittin treatment on the morphology of *mrdA* mutants

**Figure 5:**
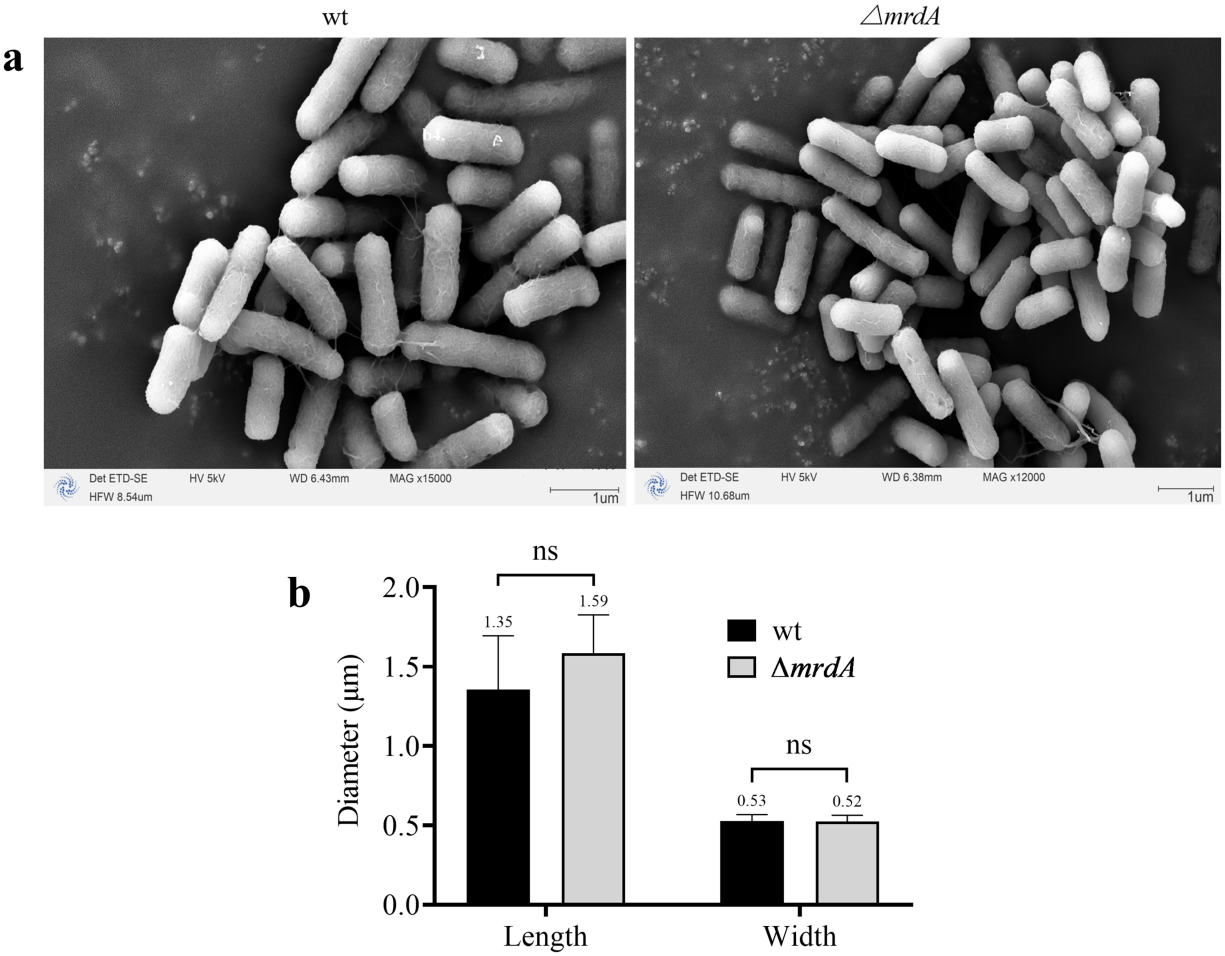
Morphology of wt and ∆*mrdA* strains under SEM **(a)** and diameter measurements **(b)** (*n* = 8).

SEM analysis revealed that both wt and ∆*mrdA* strains of *E. coli* were rod-shaped in the absence of melittin treatment (Figure 5a), and the sizes of these two strains were not significantly different (Figure 5b). The average length of wt and ∆*mrdA* strains were 1.35 μm and 1.59 μm, respectively (*p* = 0.1072), and 0.53 μm and 0.52 μm wide in diameter, respectively (*p* = 0.8895).

**Figure 6:**
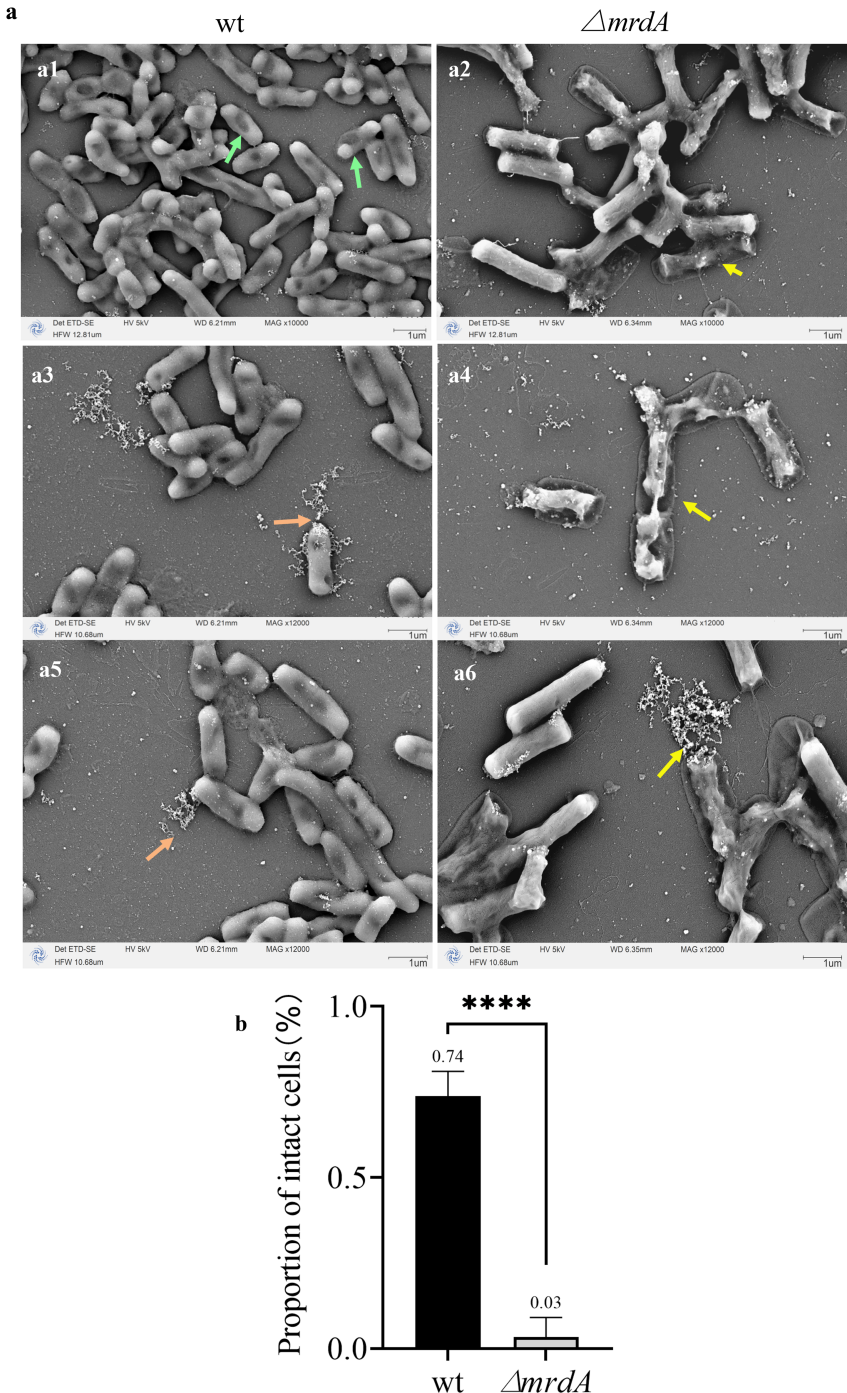
Morphological changes in wt and ∆*mrdA* strains after melittin treatment observed under SEM. **(a)** Morphological changes in wt and ∆*mrdA* strains after melittin treatment. Green arrows: After melittin treatment, localized indentations are visible on wt cells, with the weakest areas eventually rupturing, leading to the release of intracellular contents and cell death (orange arrows). In contrast, ∆*mrdA* cells exhibit no obvious indentations on their surface but instead, display widespread permeability, except at the poles, followed by multiple ruptures along the cell surface, resulting in cell lysis and death (yellow arrows). **(b)** Intactness of the wt and ∆*mrdA* cells after melittin treatment, based on SEM observations.

Both wt and ∆*mrdA* strains showed cell deformation and death following melittin treatment. However, the morphology and processes were notably distinct. Cells in the wt strain showed one or more localized pitting changes after melittin exposure (Figure 6, green arrows). The weakest depressions eventually ruptured, leading to cell death and the release of intracellular material (Figures 6a3,a5, orange arrows). In contrast, no obvious depressions were observed on the surface of ∆*mrdA* cells; instead, the entire cell became permeable except at both ends, and subsequently, multiple ruptures occurred on the cell surface and rapid cytolytic death occurred (Figures 6a2,a4,a6, yellow arrows). ∆*mrdA* deaths occurred rapidly and were more extensive compared to those in wt cells after 2 h of melittin treatment. The diffuse white highlights visible in the SEM images correspond to the intracellular effluent following bacterial death. These highlights were more pronounced in the picture background of ∆*mrdA* images compared to the wt strain images.

In addition, the proportion of intact cells treated with melittin was calculated as determined by the presence of cell rupture and cellular content leakage. As shown in Figure 6b, the average intact cell number in the wt strain was 74%, while it was only 3% in the ∆*mrdA* strain, and the latter was only 4.05% of the wt intact cell count, with a significant difference (*p* = 0.0008). These findings are consistent with the previous results from the dot-plate experiments (Figures 2c–e). It also further confirmed the speculation about the reason for the reduction of intracellular melittin content in ∆*mrdA* cells.

### 3.6 Effect of melittin on the morphology of *mrdA*-overexpressing strains

**Figure 7:**
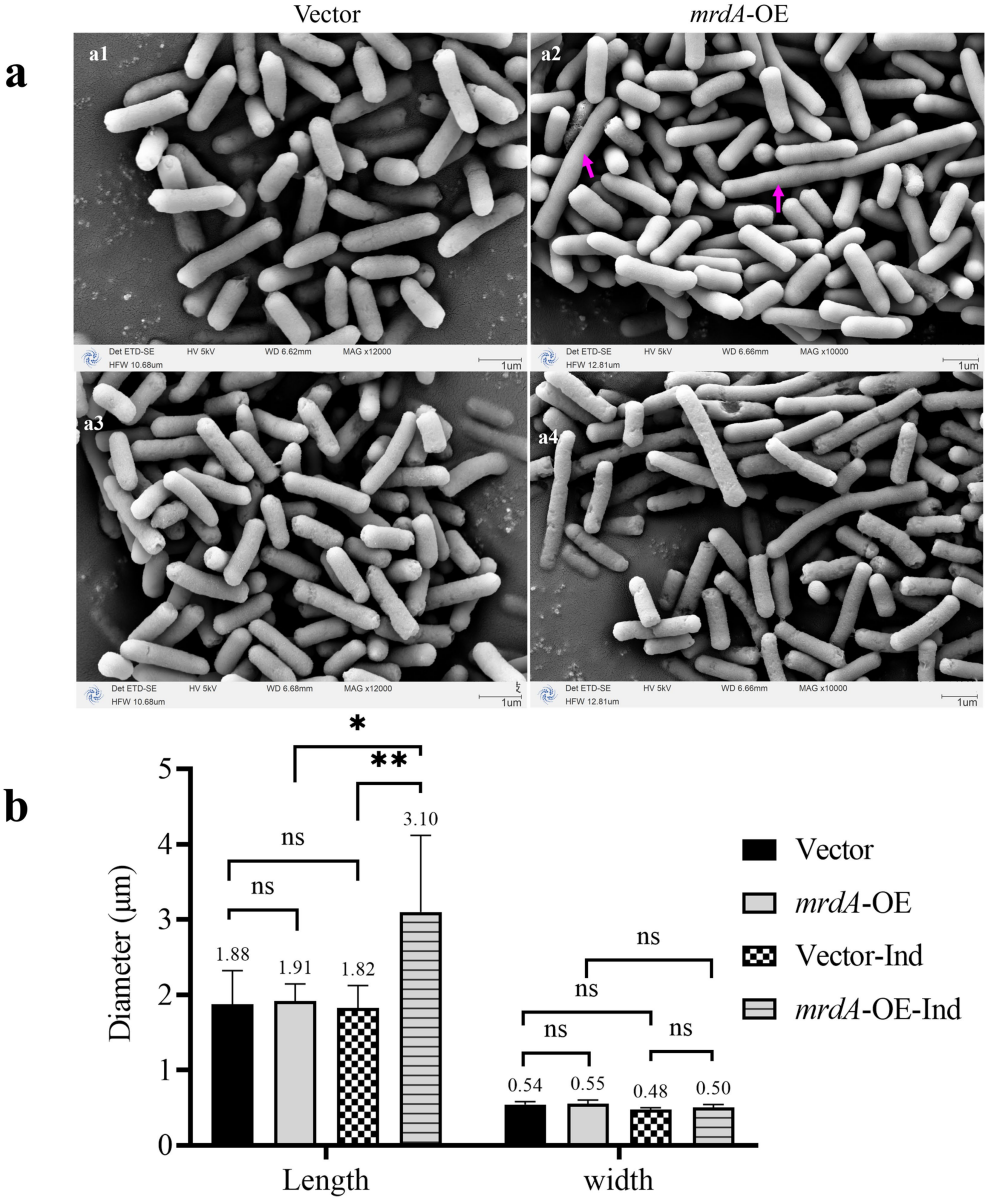
Morphology of vector and *mrdA*-OE **(a)** and diameter measurements **(b)** (*n* = 8). **(a1,a2)** Show the pre-induction morphology of vector and *mrdA*-OE strains. The cells show a significant increase in length due to leaky expression (purple arrows). **(a3,a4)** Show the post-induction morphology of vector and *mrdA*-O strains, both of which maintain a rod shape. **(b)** Diameter measurements of vector and *mrdA*-OE cells before and after induction (“Ind” indicates after induction).

Vector and *mrdA-*OE *E. coli* strains retained their rod-shaped morphology when cultured in autoinduction medium until the pre-logarithmic growth phase (OD = 0.4) (Figure 7a). There was no significant difference in the size of the two bacteria in both the length (*p* = 0.8429) and width diameters (*p* = 0.6558) (Figure 7b). It is important to note that even though the late log phase of growth was not reached and induction had not theoretically started, a very small number of *mrdA*-OE bacteria exhibited leaky expression and the bacteria became longer (Figure 7a2, as indicated by the purple arrow). These elongated cells were excluded from statistical measurements in the radial line statistics.

Both vector and *mrdA-*OE cells remained rod-shaped (Figures 7a3,a4) when cultured in autoinduction medium up to OD = 1.0 (both overexpressed and unloaded strains had been induced). However, the long diameter of *mrdA*-OE increased significantly: 1.7 times higher than that of vector after induction, and 1.62 times higher than that of *mrdA*-OE before induction (*p* = 0.0067 and *p* = 0.0116, respectively), while the width diameter did not change significantly (*p* = 0.4146 and *p* = 0.1276, respectively). For the vector strain, there was no significant difference in either the length or width diameters of the radial lines before and after induction (*p* = 0.8155 and *p* = 0.1409, respectively) (Figure 7b).

**Figure 8:**
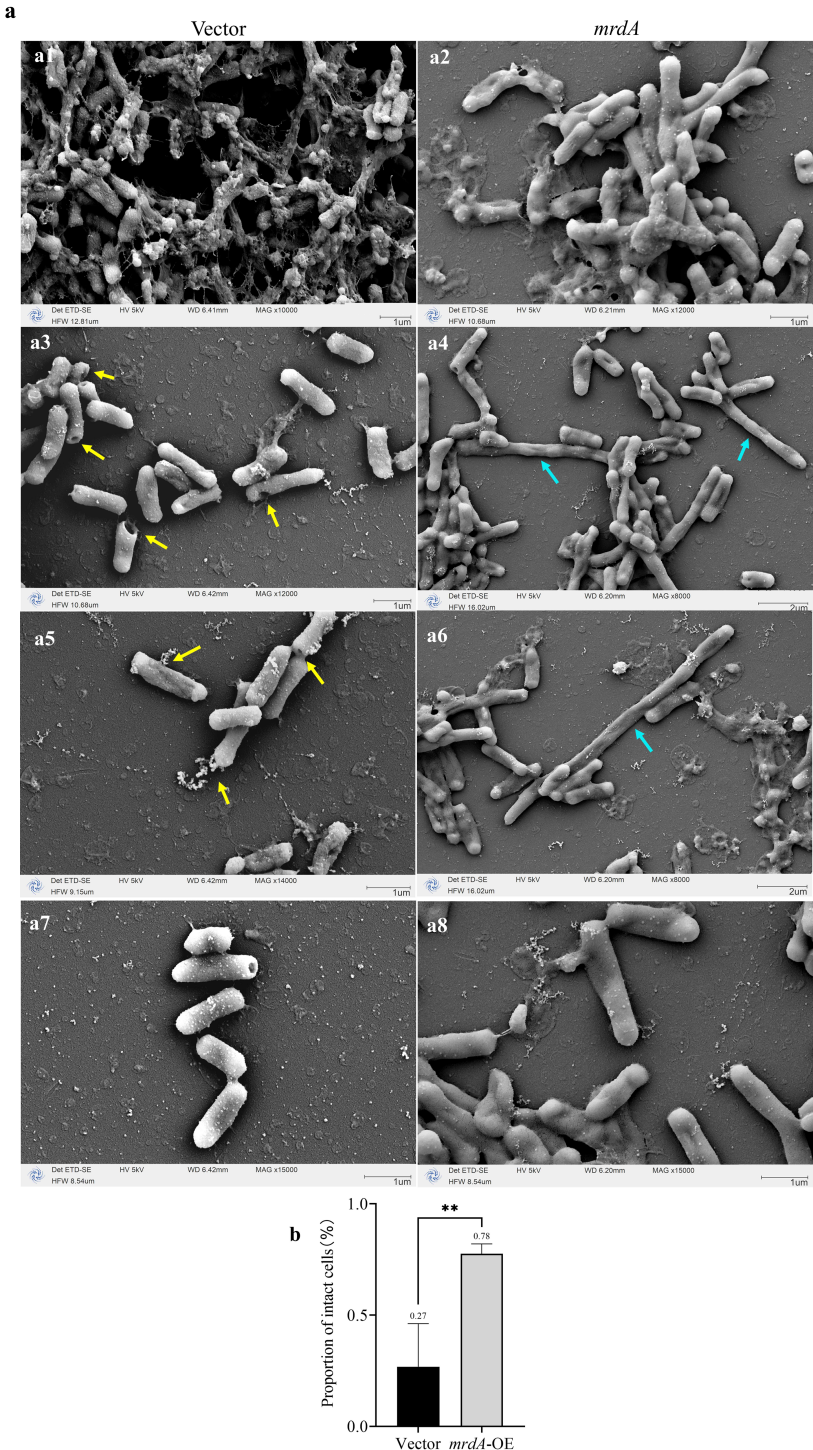
Morphological changes in vector and *mrdA*-OE cells after melittin treatment, as observed under SEM. **(a)** Morphological changes in vector and *mrdA*-OE strains after melittin treatment. Yellow arrows: vector cells develop localized pores due to melittin (GQ) exposure, with no significant morphological changes. Cyan arrows: *mrdA*-OE cells exhibit pronounced morphological changes under GQ treatment, with elongated cells showing segmental collapse and shrinkage, resulting in uneven rod-shaped forms. **(b)** Intactness of the vector and *mrdA*-OE cells after melittin treatment, as observed under SEM.

With respect to morphological responses to melittin treatment, vector cells showed significant mortality compared to *mrdA*-OE when induced strains were treated with melittin for 2 h (Figure 8). In response to melittin, vector cells showed local pore formation (Figures 8a3,a5, yellow arrows), leakage of cellular contents, and cell death, during which cell morphology changed less. In contrast, *mrdA-*OE showed marked morphological changes, with elongated cells showing segmental collapse and shrinkage, making the rod-shaped morphology uneven in thickness (Figures 8a4,a6, cyan arrows). The vector strain images also revealed more diffuse cellular contents in the background compared to *mrdA*-OE images.

The proportion of intact cells treated with melittin was further analyzed. As shown in Figure 8b, the mean number of intact cells was 27% in the vector strain and 78% in *mrdA*-OE, which was 2.89 times higher than vector cells, with a significant difference (*p*-value = 0.0022).

## 4 Discussion

The *mrdA* gene in *E. coli* encodes a transpeptidase that plays a crucial role in the cross-linking of amino acids in different linear chains of amino sugars, resulting in the formation of a robust and rigid three-dimensional structure of peptidoglycan. Neelay et al. (2017) found that a combination of amoxicillin and melittin increased the susceptibility of *Klebsiella pneumoniae* to amoxicillin. They speculated that the binding of amoxicillin to bacterial transpeptidase (commonly known as penicillin-binding proteins) interferes with peptidoglycan synthesis. This facilitates melittin’s entry into the bacterial cell, which then exerts a bactericidal effect.

In our study, the gene encoding transpeptidase *mrdA* of *E. coli* was knocked out and heterologously expressed. We found that the degree of peptidoglycan cross-linking (or rigidity) was directly proportional to the resistance of host bacteria to melittin and inversely proportional to the intracellular melittin content. These results confirm the barrier effect of peptidoglycan on antimicrobial peptides. This effect appears to be largely physical in nature. As shown in previous studies, Gram-positive bacteria are generally less sensitive to antimicrobial peptides than Gram-negative bacteria, primarily due to the thickness of peptidoglycan in the former, which is 3 to 10 times thicker than that found in Gram-negative bacteria (Schurr et al., 2022). In addition, the pores of peptidoglycan shrink as peptidoglycan thickness increases (Neelay et al., 2017). This reduction in pore size may explain why highly cross-linked or rigid peptidoglycan can more effectively prevent melittin from entering the host bacterial cell.

It has been proposed in previous studies that melittin and cecropin A may bind to peptidoglycan as well as lipopolysaccharide *in vitro* (Neelay et al., 2017; Park et al., 2006). However, the exact mechanism and mode of binding to peptidoglycan are poorly understood. In this study, we further demonstrated that peptidoglycan with a high degree of cross-linking exhibited a stronger binding adsorption capacity for melittin. However, it is unclear whether melittin binds to peptidoglycan *in vivo*, and whether such binding disrupts the structure of the peptidoglycan layer, triggering or promoting bacterial death.

Henk et al. (1995) investigated the effects of melittin on *E. coli* at bactericidal concentrations using transmission and scanning electron microscopy. They found that melittin treatment resulted in fundamental destruction of the outer membrane, inner membrane, and peptidoglycan layer. However, it was not indicated in their study whether this effect of a peptidoglycan structural change was directly caused by melittin binding to peptidoglycan or an indirect consequence to factors such as changes in intracellular osmotic pressure or interference with the peptidoglycan synthesis process, resulting in fragmentation of the peptidoglycan layer. However, the results of our study showed that although melittin demonstrated a binding affinity to peptidoglycan, it remains uncertain whether this interaction alone exerts a direct destructive effect on peptidoglycan structure. Nevertheless, the findings suggest that melittin’s binding—whether destructive or not—could not counteract the protective effects of increased peptidoglycan cross-linking in enhancing bacterial resistance.

It has been proposed that in *mrdA*-depleted *E. coli*, due to reduced peptidoglycan cross-linking, the rod-shaped morphology changes to a spherical shape under the influence of intracellular osmotic pressure and the cells subsequently undergo lysis and death with prolonged culture (Ogura et al., 1989). However, it has been shown in other studies that *mrdA*-depleted cells can still grow and multiply stably under specific conditions. These conditions include a concomitant increase in essential cell division proteins like FtsQ, FtsA and FtsZ (Navarro et al., 1998), a slower rate of cell growth (Barbour et al., 1981), and elevated levels of ppGpp (guanosine 3′-diphosphate 5′-diphosphate) above a certain threshold value (Joseleau-Petit et al., 1994).

On the other hand, there are different conclusions about the morphological changes in *mrdA*-overexpressing strains. Tropini et al. (2014) observed that *E. coli* overexpressing *mrdA* was morphologically indistinguishable from unloaded strains, while El-Hajj and Newman (2015) observed varying degrees of elongation in *mrdA*-overexpressing *E. coli*. In our study, we did not find differences in morphology, growth rate, or reproductive capacity in *mrdA-*deleted *E. coli* MG1655 compared to wild type strains. Interestingly, peptidoglycan extraction from each strain revealed that the peptidoglycan content of the mutant strain was only 16.7% of that of the wild type at the same amount of bacteria, while the bacterial rod-shaped morphology did not change. The reason why the rod-shaped morphology was still maintained in the mutant strain when only 1/6th of the peptidoglycan content was presented warrants further study.

On the other hand, we found different degrees of elongation in the morphology of the *mrdA-*overexpressing *E. coli* MG1655 strain as compared to unloaded strains. This elongation is presumably related to increased transpeptidase activity, which continuously incorporates peptidoglycan monomers into existing peptidoglycan chains, resulting in simultaneous peptidoglycan thickening and cell elongation. Peptidoglycan has been reported to have some elasticity, and it has been found that isolated peptidoglycan sacculus from *E. coli* can deform to three times their original area under the tensile force without rupturing, and the deformation along the long axis is two to three times greater than that along the short axis (Koch and Woeste, 1992). This explains the observed elongation of the *mrdA*-overexpressing strain in the present study in terms of length but not width.

In response to melittin treatment, the *mrdA* mutant cells, unlike the wild-type strain, did not produce significant localized deformation but exhibited lytic rupture of multiple cells. This suggests that the overall rigidity of the cell wall in the mutant strain became fragile due to the absence of peptidoglycan cross-linking. In contrast, the *mrdA*-overexpressing bacteria, although elongated and potentially binding more melittin to their outer membrane lipopolysaccharide, experience melittin resistance. This melittin resistance in overexpressed strains is likely due to their thickened peptidoglycan layer, which acts as a barrier, preventing melittin from penetrating the cells and exerting its bactericidal effects.

In this study, we revealed for the first time the key role of the *mrdA* gene in conferring melittin resistance in *E. coli* and extensively investigated the mechanistic relationship between peptidoglycan cross-linking and antimicrobial resistance. The findings indicate that increased peptidoglycan cross-linking, mediated by *mrdA* expression, plays a crucial role in melittin resistance. However, these conclusions in this study are drawn from *in vitro* experiments using the *E. coli* MG1655 strain. Therefore, the actual resistance effect of peptidoglycan cross-linking requires further validation through *in vivo* models. Additionally, an in-depth structural analysis of peptidoglycan modifications is essential for a better understanding of the mechanism of action of antimicrobial peptides like melittin on bacterial cell walls. The *mrdA* gene’s influence on peptidoglycan cross-linking may not be limited to melittin resistance. Given the structural role of peptidoglycan in bacterial cell walls, alterations in *mrdA* expression could potentially affect susceptibility to a broad range of antimicrobial peptides that target or interact with peptidoglycan. This broader impact on resistance could have significant implications for the development of new antimicrobial agents. Our study on the relationship between the *mrdA* gene and melittin resistance in *E. coli* has two main limitations. Firstly, the experiments were primarily conducted under *in vitro* conditions using the single *E. coli* MG1655 strain, which limits the generalizability of the findings. Future studies will need to further validate the impact of peptidoglycan cross-linking on melittin resistance using *in vivo* models. Secondly, while the study reveals the role of the *mrdA* gene in peptidoglycan cross-linking and its association with melittin resistance, the specific molecular mechanisms of the interaction between melittin and peptidoglycan require further exploration. Future work could utilize techniques such as molecular dynamics simulations, crystallography, and mass spectrometry to more detailedly elucidate the molecular basis of these interactions.

## 5 Conclusion

Our research has revealed that the augmentation of peptidoglycan cross-linking in *E. coli* is associated with increased resistance to melittin. The underlying mechanism we propose is that heightened cross-linking of peptidoglycan leads to a thicker cell wall and a reduction in pore size within the cell wall. Such structural modifications potentially diminish melittin’s capacity to inflict damage on the cell wall and impede its penetration into the bacterial cell. Consequently, these alterations bestow upon the bacteria a heightened level of resistance to melittin.

## Data Availability

The data presented in the study are readily available at the below: *mrdA* - Peptidoglycan D, D-transpeptidase MrdA - *Escherichia coli* (strain K12) | UniProtKB | UniProt and the NCBI with accession number: X04516.1.

## References

[ref1] Alkasher I. Badi W. (2020). Gram-positive vs gram-negative.

[ref2] Arthur M. Courvalin P. (1993). Genetics and mechanisms of glycopeptide resistance in enterococci. Antimicrob. Agents Chemother. 37, 1563–1571. doi: 10.1128/AAC.37.8.1563, PMID: 8215264 PMC188020

[ref3] Barbour A. G. Mayer L. W. Spratt B. G. (1981). Mecillinam resistance in *Escherichia coli*: dissociation of growth inhibition and morphologic change. J. Infect. Dis. 143, 114–121. doi: 10.1093/infdis/143.1.114, PMID: 6260864

[ref4] Blanco P. Hjort K. Martínez J. L. Andersson D. I. (2020). Antimicrobial peptide exposure selects for resistant and fit *Stenotrophomonas maltophilia* mutants that show cross-resistance to antibiotics. mSphere 5:e00717. doi: 10.1128/msphere.00717-20 32999081 PMC7529437

[ref5] Brewster J. D. (2003). A simple micro-growth assay for enumerating bacteria. J. Microbiol. Methods 53, 77–86. doi: 10.1016/S0167-7012(02)00226-9, PMID: 12609726

[ref6] Brötz H. Bierbaum G. Leopold K. Reynolds P. E. Sahl H. G. (1998). The lantibiotic mersacidin inhibits peptidoglycan synthesis by targeting lipid II. Antimicrob. Agents Chemother. 42, 154–160. doi: 10.1128/AAC.42.1.154, PMID: 9449277 PMC105472

[ref7] El-Hajj Z. W. Newman E. B. (2015). An *Escherichia coli* mutant that makes exceptionally long cells. J. Bacteriol. 197, 1507–1514. doi: 10.1128/JB.00046-15, PMID: 25691528 PMC4372735

[ref8] Fisher J. F. Meroueh S. O. Mobashery S. (2005). Bacterial resistance to β-lactam antibiotics: compelling opportunism, compelling opportunity. Chem. Rev. 105, 395–424. doi: 10.1021/cr030102i, PMID: 15700950

[ref9] Guilhelmelli F. Vilela N. Albuquerque P. Derengowski Lda S. Silva-Pereira I. Kyaw C. M. (2013). Antibiotic development challenges: the various mechanisms of action of antimicrobial peptides and of bacterial resistance. Front. Microbiol. 4:353. doi: 10.3389/fmicb.2013.00353 24367355 PMC3856679

[ref10] Habets M. G. Brockhurst M. A. (2012). Therapeutic antimicrobial peptides may compromise natural. Biol. Lett. 8, 416–418. doi: 10.1098/rsbl.2011.1203 22279153 PMC3367763

[ref11] Hébert L. Courtin P. Torelli R. Sanguinetti M. Chapot-Chartier M. P. Auffray Y.. (2007). *Enterococcus faecalis* constitutes an unusual bacterial model in lysozyme resistance. Infect. Immun. 75, 5390–5398. doi: 10.1128/IAI.00571-07, PMID: 17785473 PMC2168276

[ref12] Henk W. G. Todd W. J. Enright F. M. Mitchell P. S. (1995). The morphological effects of two antimicrobial peptides, hecate-1 and melittin, on *Escherichia coli*. Scanning Microsc. 9:19. 8714745

[ref13] Hsu S.-T. D. Breukink E. Tischenko E. Lutters M. A. G. de Kruijff B. Kaptein R.. (2004). The nisin–lipid II complex reveals a pyrophosphate cage that provides a blueprint for novel antibiotics. Nat. Struct. Mol. Biol. 11, 963–967. doi: 10.1038/nsmb830, PMID: 15361862

[ref14] Joseleau-Petit D. Thévenet D. D'Arl R. (1994). ppGpp concentration, growth without PBP2 activity, and growth-rate control in *Escherichia coli*. Mol. Microbiol. 13, 911–917. doi: 10.1111/j.1365-2958.1994.tb00482.x, PMID: 7815948

[ref15] Koch A. L. Woeste S. (1992). Elasticity of the sacculus of *Escherichia coli*. J. Bacteriol. 174, 4811–4819. doi: 10.1128/jb.174.14.4811-4819.1992, PMID: 1624468 PMC206280

[ref16] Kubicek-Sutherland J. Z. Lofton H. Vestergaard M. Hjort K. Ingmer H. Andersson D. I. (2016). Antimicrobial peptide exposure selects for *Staphylococcus aureus* resistance to human defence peptides. J. Antimicrob. Chemother. 72, 115–127. doi: 10.1093/jac/dkw381, PMID: 27650186 PMC5161045

[ref17] Mousavi Maleki M. S. Sardari S. Ghandehari Alavijeh A. Madanchi H. (2023). Recent patents and FDA-approved drugs based on antiviral peptides and other peptide-related antivirals. Int. J. Pept. Res. Ther. 29:5. doi: 10.1007/s10989-022-10477-z, PMID: 36466430 PMC9702942

[ref18] Navarro F. Robin A. D’Ari R. Joseleau-Petit D. (1998). Analysis of the effect of ppGpp on the ftsQAZ operon in *Escherichia coli*. Mol. Microbiol. 29, 815–823. doi: 10.1046/j.1365-2958.1998.00974.x, PMID: 9723920

[ref19] Neelay O. P. Peterson C. A. Snavely M. E. Brown T. C. TecleMariam A. F. Campbell J. A.. (2017). Antimicrobial peptides interact with peptidoglycan. J. Mol. Struct. 1146, 329–336. doi: 10.1016/j.molstruc.2017.06.018

[ref20] Ogura T. Bouloc P. Niki H. D'Ari R. Hiraga S. Jaffé A. (1989). Penicillin-binding protein 2 is essential in wild-type *Escherichia coli* but not in *lov* or *cya* mutants. J. Bacteriol. 171, 3025–3030. doi: 10.1128/jb.171.6.3025-3030.1989, PMID: 2656638 PMC210010

[ref21] Olaitan A. O. Thongmalayvong B. Akkhavong K. Somphavong S. Paboriboune P. Khounsy S.. (2015). Clonal transmission of a colistin-resistant *Escherichia coli* from a domesticated pig to a human in Laos. J. Antimicrob. Chemother. 70, 3402–3404. doi: 10.1093/jac/dkv252, PMID: 26283671

[ref22] Park S.-C. Kim J. Y. Shin S. O. Jeong C. Y. Kim M. H. Shin S. Y.. (2006). Investigation of toroidal pore and oligomerization by melittin using transmission electron microscopy. Biochem. Biophys. Res. Commun. 343, 222–228. doi: 10.1016/j.bbrc.2006.02.090, PMID: 16540094

[ref23] Roland K. L. Martin L. E. Esther C. R. Spitznagel J. K. (1993). Spontaneous pmrA mutants of *Salmonella typhimurium* LT2 define a new two-component regulatory system with a possible role in virulence. J. Bacteriol. 175, 4154–4164. doi: 10.1128/jb.175.13.4154-4164.1993, PMID: 8391535 PMC204845

[ref24] Samuelsen Ø. Haukland H. H. Jenssen H. Krämer M. Sandvik K. Ulvatne H.. (2005). Induced resistance to the antimicrobial peptide lactoferricin B in *Staphylococcus aureus*. FEBS Lett. 579, 3421–3426. doi: 10.1016/j.febslet.2005.05.017, PMID: 15946666

[ref25] Schaub R. E. Dillard J. P. (2017). Digestion of peptidoglycan and analysis of soluble fragments. Bio Protoc. 7:e2438. doi: 10.21769/BioProtoc.2438, PMID: 28932761 PMC5602577

[ref26] Schurr A. Barker L. Volle C. B. (2022). Characterizing the roll of the cell wall in pore formation by antimicrobial peptides. Biophys. J. 121:218a. doi: 10.1016/j.bpj.2021.11.1621

[ref27] Studier F. W. (2014). “Stable expression clones and auto-induction for protein production in *E. coli*” in Structural genomics. Methods in molecular biology (Totowa, NJ: Humana Press), 17–32. 10.1007/978-1-62703-691-7_2 24203322

[ref28] Torres R. T. Cunha M. V. Araujo D. Ferreira H. Fonseca C. Palmeira J. D. (2021). Emergence of colistin resistance genes (*mcr-1*) in *Escherichia coli* among widely distributed wild ungulates. Environ. Pollut. 291:118136. doi: 10.1016/j.envpol.2021.118136, PMID: 34530238

[ref29] Tropini C. Lee T. K. Hsin J. Desmarais S. M. Ursell T. Monds R. D.. (2014). Principles of bacterial cell-size determination revealed by cell-wall synthesis perturbations. Cell Rep. 9, 1520–1527. doi: 10.1016/j.celrep.2014.10.027, PMID: 25456140 PMC4254626

[ref30] Wiegand I. Hilpert K. Hancock R. E. (2008). Agar and broth dilution methods to determine the minimal inhibitory concentration (MIC) of antimicrobial substances. Nat. Protoc. 3, 163–175. doi: 10.1038/nprot.2007.521, PMID: 18274517

[ref31] Zhao C. Yan S. Luo Y. Song Y. Xia X. (2024). Analyzing resistome in soil and human gut: a study on the characterization and risk evaluation of antimicrobial peptide resistance. Front. Microbiol. 15:1352531. doi: 10.3389/fmicb.2024.1352531, PMID: 38591036 PMC10999558

