# Supplementary material for: Functional analysis of the Escherichia coli mrdA gene in melittin resistance
Source: Front Microbiol. 2025 Mar 5;15:1516808. doi: 10.3389/fmicb.2024.1516808 (PMC11920165; doi:10.3389/fmicb.2024.1516808)
Supplement: Supplementary file 1 [file Data_Sheet_1.docx]

**Supplementary table 1** Primers related to *mrdA* gene knockout

| Primer | sequence |
| --- | --- |
| gRNA1 | gatgaagctacatcagctctgga |
| gRNA2 | gaatggccatgaccaaaatcccttatcagaaaagccgtcttcctttgtgtag |
| mrdA-F | ggtttttcacatgggctcacacacatcggctgtttaaccgggttactcttctg |
| mrdA-R | cagaagagtaacccggttaaacagccgatgtgtgtgagcccatgtgaaaaacc |
| mrdA-R1 | cgctcagtggaacgaaaactcacgtgtaacagtcattgcgatattagaatc |
| left arm（991 bp） | ggcagaataccgcttaccataccaatatttacgaagacataaacgaataatatcagcattaagccgccagccatgacgcgaccaaaggtggtttgcgctctggcggctatccacagcccgcgcatgatcagcagaatgtagagagcgagcagaatcagaatgcccactaatcccagctcttccgccagtaccgcgaagataaagtcagtatggcgttcggggagaaattcaagctgtgactgagtgccgtgcagccagcctttgccgcgtaatccgccggagccaatagcaattttagactgaataatgtgatagcccgcgccgagtgggtctgattccgggtccaggagcatcattacgcgctggcgctggtaatcatgcatcaggaagaaccacagaatcggaatgaacgccgctaccagcactactgcgacgccaatcagacgccagctaaggccagagaggaacagtacaaacagaccggaaagcgcaacgaggattgatgttcccaggtcaggctgtgcagccaccagcagcgtgggcataaatatcagcaccagcgcgatgccagtgttcttcaacgatggcgggcaaacgtcgcggttgataaagcgcgcaaccatcagtggtacggctattttggcaatttccgacggctgaaaacgaacaataccgaggtccagccagcgttgagcacctttagagatggcaccgaaagcatctaccgccaccagcaaaataatacagatgatatagagatagggggcccagccttcataaacgcgtggaggaatttgcgccatcaccaccatgatgaccagacccatcgcgatttggccgattttacgctccatcatgccaatatcctgaccgctggcgctccagataaccagggcgctgtaaaccagcaatgccagtaagatcagcagcattgtgggatcgagatggactttatcccagaatgtttttttattcggattatccgtcatga |
| right arm（1008 bp） | ccgctgcgttttctactcaaagctcccttatcactcacggtgataaggatggttggtggtgatgctccacgcccggtacagactctctgcgaccagcacgcgaaccagcggatgggggagggtaagcgccgacagcgaccagctctgctcagccgccgctttacaggcaggcgacaacccttcaggcccgccaatcagtagactgacgtcgcgaccatccagcttccagcgttccagctcagcggctaactgcggcgtatcccagggcttgcctggaatatcgagggtgacaatgcggtttttgcctgcggccgccaacatctgctcaccctctttgtcgagtatgcgcttgatgtccgcattcttgccgcgttttccggccggaatttcaatcagctcgaagggcatatctttcggaaaacgacgcaggtactcggtaaaaccggtttgtacccagtccggcattttcgttcccacggcgacaagttgcagcttcacgcattaactccagagtttttccagttcatacaggcgacggctctcttcctgcatgacatggacaatcacatcgcccaaatccacgacaatccagtcggcgctgttttcaccttctacgccgagcggtaacaggcccgctgcgcgagactcctgcacaacgtggtcagcaatggacataacatgacggctggacgtacccgtacagatgatcatgcagtcggtgatgctggatttgccctgaacgtctaaggcgatgatgtcctgacctttgaggtcatcaattttgtcgataacaaaatcctggagtgctttaccctgcaagttttccccctgggtgaatcaaatagataaaaatggtctgtcagtatacctgaaccagaggcgatttcgggacaattgtcgccgaatcggctttcgaaagtgggctatcatcccaccccgcgccgcagattgcatcgccatttttgtaaaacaatttctacaaagtcgtgtctggcggaaaaagtctgg |

**Supplementary table 2** Primers related to the construction of the pET28a-*mrdA* vector

| Primer | sequence |
| --- | --- |
| mrdA-CDS FP | atgaaactacagaactcttttcgcgact |
| mrdA-CDS RP | atggtcctccgctgcg |
| mrdA FP | ccgcaagcttgtcgacatggtcctccgctgcg |
| mrdA RP | tcgcggatccgaattcatgaaactacagaactcttttcgcgac |

**Supplementary table 3** Auto-induction culture medium storage solution

| storage solution | Composition |
| --- | --- |
| ZY | Tryptone1%(v/w) |
|  | Yeast extract 0.5% (v/w) |
| 50xM* | 1.25 M Na_2_HPO_4_ |
|  | 1.25 M KH_2_PO_4_ |
|  | 2.5 M NH_4_Cl |
|  | 0.25 M Na2SO4 |
| 50x5052* | Glycerol 25%(v/w) |
|  | Glucose2.5%(v/w) |
|  | Alpha-lactose10% (v/w) |
| 1 M MgSO_4_ | MgSO4·7H2O |
| 50 mM FeCl_3_* | FeCl_3_ |

*0.22 μ m membrane filtration for sterilization

**Supplementary table 4** Auto-induction culture medium working solution

| storage solution | usage（mL） |
| --- | --- |
| ZY | 968 |
| 50x5052 | 20 |
| 50xM | 20 |
| 1 M MgSO_4_ | 2 |
| FeCl_3_ | 0.2 |

Note：The high phosphate concentration of self induced culture medium requires the addition of kanamycin (≥ 100 μ g/mL) at a concentration two times or more higher than that of ordinary LB medium

**Supplementary table 5** Ultrasonic buffer solution

| storage solution | Required quantity (prepare 1 L) | final concentration |
| --- | --- | --- |
| Tris-HCl（1 M，pH 8.0） | 50 mL | 50 mM |
| NaCl | 29.22 g | 500 mM |
| glycerol | 150 mL | 15%（V/V） |
| PMSF* | 10 mL（100 mM） | 1 mM |
| DTT* | 154 mg | 1 mM |
| EDTA* | 150 mg | 0.5 mM |
| lysozyme* | 1 g | 1 mg/mL |

*Add before use

**Supplementary table 6** Conditions for HPLC analysis for GQ

| Solvent | Preparation Method | Parameters at Use |
| --- | --- | --- |
| Solvant A | 0.1% TFA (Trifluoroacetic acid) + Acetonitrile | 38% |
| Solvant B | 0.1% TFA+H_2_O | 62% |

**Supplementary table 7** Solution preparation for peptidoglycan extraction

| Solvent | Preparation Method |
| --- | --- |
| PB（25 mM phosphate buffer pH=6） | 0.2973%(w/v)NaH_2_PO_4_·H_2_O；0.0926%(w/v)Na_2_HPO_4_·7H_2_O；Confirm pH=6，autoclave |
| PB+8%(w/v) SDS | 0.2973% (w/v)NaH_2_PO_4_·H_2_O；0.0926%(w/v)Na_2_HPO_4_·7H_2_O；8.0% (w/v) SDS |
| PBS | 137 mM NaCl；2.7 mM KCl；10 mM Na_2_HPO_4_；  1.8 mM KH_2_PO_4_；autoclave |


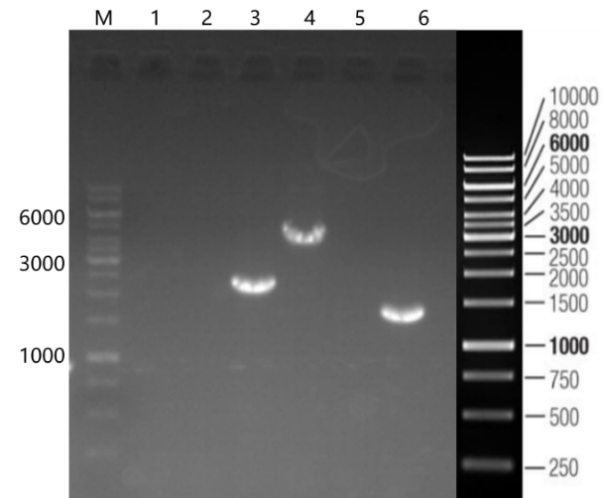


**Supplementary figure 1：** Identification of *mrdA* gene knockout by colony PCR

M: DL10000; 1–2: H_2_O; 3: PCR result with ∆mrdA-mrdA-F/mrdA-R primers; 4: PCR result with wt-mrdA-F/mrdA-R primers; 5: PCR result with ∆mrdA-mrdA-F/mrdA-R1 primers; 6: PCR result with wt-mrdA-F/mrdA-R1 primers.

The results are shown in Supplementary figure 1, with colony PCR confirming the successful knockout of the *mrdA* gene in the ∆*mrdA* strain. The PCR product from primers at both ends of the left and right arms in ∆*mrdA* strain was 2402 bp, as seen in lane 3. The wild-type strain produced a 4289 bp product, as shown in lane 4. For the ∆*mrdA* strain, PCR with primers from the left arm and the middle region of the *mrdA* gene resulted in a 0 bp product, indicating successful knockout, as shown in lane 5. The wild-type strain (wt) produced a 1795 bp product, as seen in lane 6.
